# Influence of quaternary cation compound on the size of the *Escherichia coli* small multidrug resistance protein, EmrE

**DOI:** 10.1016/j.bbrep.2018.02.001

**Published:** 2018-02-20

**Authors:** S. Junaid S. Qazi, Raymond J. Turner

**Affiliations:** Department of Biological Sciences, Faculty of Science, University of Calgary, Calgary, Alberta, Canada T2N 1N4

**Keywords:** Small multidrug resistance (SMR) protein, EmrE, Integral membrane protein folding, Quaternary ammonium compounds (QAC), Quaternary cation compounds (QCC), Ligand binding, Hexahistidine (His_6_) tag, Structural plasticity, Dynamic light scattering

## Abstract

EmrE is a member of the small multidrug resistance (SMR) protein family in *Escherichia coli*. It confers resistance to a wide variety of quaternary cation compounds (QCCs) as an efflux transporter driven by the transmembrane proton motive force. We have expressed hexahistidinyl (His_6_) – myc epitope tagged EmrE, extracted it from membrane preparations using the detergent n-dodecyl-β-D-maltopyranoside (DDM), and purified it using nickel-affinity chromatography. The size of the EmrE protein, in DDM environment, was then examined in the presence and absence of a range of structurally different QCC ligands that varied in their chemical structure, charge and shape. We used dynamic light scattering and showed that the size and oligomeric state distributions are dependent on the type of QCC. We also followed changes in the Trp fluorescence and determined apparent dissociation constants (*K*_d_). Overall, our in vitro analyses of epitope tagged EmrE demonstrated subtle but significant differences in the size distributions with different QCC ligands bound.

## Highlights

• Chemical shape of ligand has significant affect on binding.
• Shape of the ligand affects the multimeric state of EmrE.
• Binding affinities strongly depend upon the ligand shape.
• EmrE shows high plasticity of structure to accommodate a wide range of ligands.

## Graphical abstract

**Figure:**
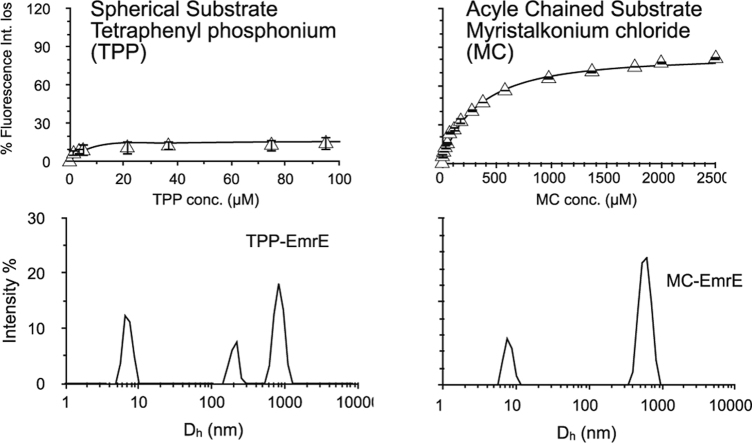

## 1 Introduction

Bacterial antiseptic and antibiotic resistance present major challenges in controlling infection, particularly in healthcare settings [1], [2], [3], [4]. Bacterial resistance to antiseptics and antibiotics can be mediated by multidrug resistance transporters [1], [2]. The *Escherichia coli* small multidrug resistance (SMR) transporter family member EmrE is an integral membrane protein comprising 110 amino acid residues and is considered to be an archetypical member of the SMR family [5], [6]. EmrE is one of many transporters responsible for antiseptic drug resistance in bacteria, and catalyzes the efflux of quaternary cationic compounds (QCCs) [7], [8]. It comprises 4 transmembrane alpha helices, connected by short loops (as reviewed by [6]), and has been shown to exist as a monomer, dimer, trimer, tetramer or even higher ordered multimers/ complexes, but the minimal functional unit is considered to be a dimer [9], [10], [11], [12], [13].

A widely used purification method for EmrE involves the use of a hexahistidine (His_6_) tag at its C-terminus, which facilitates protein purification using Ni^2+^-affinity chromatography [14]. This His_6_ tag is usually left on the protein in most structural analyses (as reviewed by [6]). However, it is reported that the precise location of the His_6_ on a protein can influence its structure and activity [15]. Almost all in vitro studies on EmrE have utilized a C-terminal fusion tag that includes a Myc epitope before the His_6_ tag (EmrE-Myc-His_6_). The Myc tag functions as a spacer that improves accessibility of the His_6_ tag, resulting in higher purification yields [9]. As a result, EmrE-Myc-His_6_ has been used in most in vitro studies of EmrE over the past two decades [16]. Because of this extensive use of a tagged version of EmrE protein by other research groups and its use in most of the structural biology studies, it is very important to explore the ligand binding behavior of this version of EmrE protein with respect to the wide range of QCC ligand substrates.

**Fig. 1:**
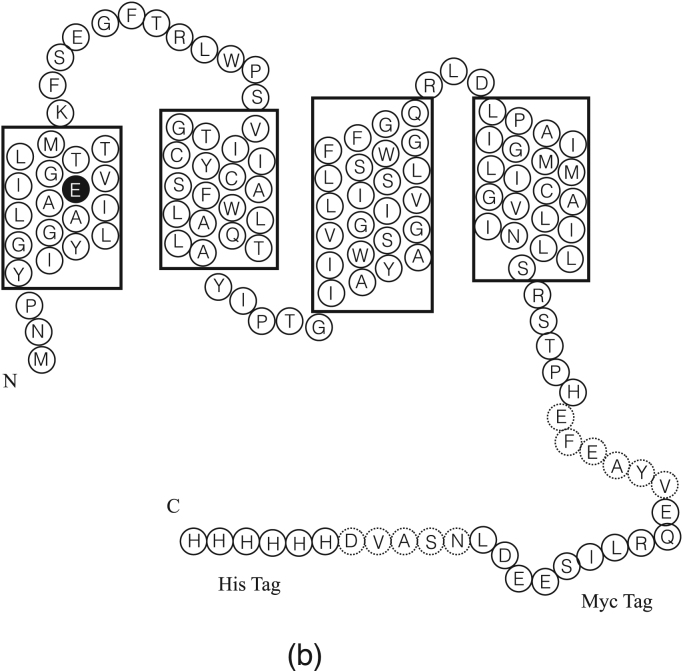
EmrE amino acid sequence that was examined in this study. Boxes indicate the predicted transmembrane regions for the protein.

The primary sequence of EmrE-Myc-His_6_ is shown in Fig. 1 [6]. High-resolution 3D analysis using cryo-electron microscopy and X-ray crystallography have reported on the conformational state of the topologically asymmetric dimer of EmrE [12], [17], [18]. NMR spectroscopy has also confirmed the presence of EmrE homodimers, wherein the putative active site involves Glu14 and residues from each monomer in structurally inequivalent environments [19], [20], [21], [22], [23]. In addition to Glu14, aromatic residues have been shown to play a role in defining the active site: for example, Trp63 was demonstrated to be located close to the QCC binding pocket, and a variant of this residue abolished ligand binding [8], [24]. Two other tryptophans are located within transmembrane segments 2 and 3 and a fourth tryptophan can be found in loop 2, all of which are expected to be involved in ligand interaction and can be used as spectroscopic probes of ligand binding [25].

Many structural variations of QCC antiseptics exist, and we have grouped them into categories of: sphere forming (based on space filling structure), polyaromatic, acyl-chained, and poly-charged. Of these, tetraphenylphosphonium chloride (TPP) has been the most commonly used in structural studies to analyze interactions within EmrE [9], [17], [26], [27], [28]. However, due to variations in experimental design, a wide range of binding affinities have been reported (Table 1). It is therefore valuable to evaluate the binding of a range of structurally distinct QCCs substrates to EmrE within a single study.

**Table 1:** Binding affinities of tetraphenyl phosphonium (TPP) to epitope tagged EmrE evaluated under different conditions from previous studies.

**Assaying condition**	**Experiment**	**Ligand**	**K**_**d**_**(μM)**	**Reference**
.08% w/v DDM	Equilibrium dialysis	[^3^H] TPP	.01 ± 0.003	[9]
0.8% w/v DDM	Saturation binding assay	[^3^H] TPP	.0028 ± 0.001	[25]
0.1% w/v DDM	Saturation binding assay	[^3^H] TPP	.0026 ± 0.0004	[17]
0.5% w/v DDM (delipidated EmrE)	Saturation binding assay	[^3^H] TPP	2.5 ± 0.5	[26]
0.5% w/v DDM (non-delipidated EmrE)			10 ± 2	

Here we present the binding of 19 QCCs with varied chemical structures and charge (structures shown in Fig. 3 as insets). The form of the protein used was purified in the presence of the detergent n-dodecyl-β-D-maltopyranoside (DDM) and contains a C-terminal Myc epitope followed by a His_6_ tag. Thus our primary question, is there a size/multimerization change with ligand binding and is this dependent on the QCC ligand type? Previous research has suggested this, but the substrate profile was small, leading us to pursue a more systematic study. Purified His_6_-myc-EmrE was evaluated using dynamic light scattering (DLS) and fluorescence spectroscopy techniques. DLS was used to compare the size distributions and multimeric state of the protein. Quenching of endogenous Trp fluorescence upon QCC binding was used to evaluate binding site affinity. We show that there are real and significant differences in how the protein responds to binding by each of the nineteen QCC ligands.

**Fig. 2:**
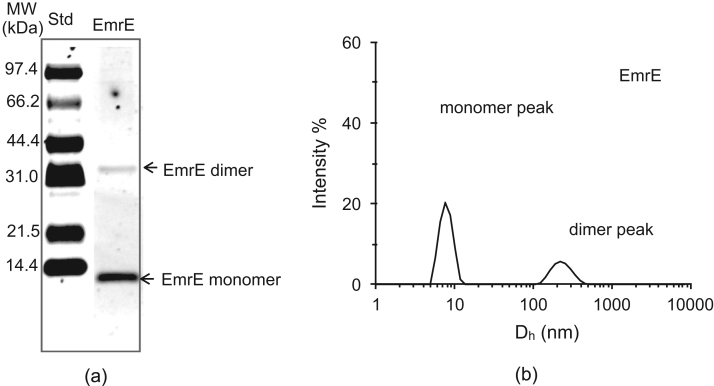
(a) SDS-T-PAGE analysis (12% acrylamide gel) of 0.08% w/v DDM purified EmrE, 1.4 µg of the protein was loaded in the respective lane. Low molecular weight (LMW) Bio-Rad protein standards were used to estimate protein weights and relative band intensity. The presence of monomeric and dimeric state is indicated for EmrE by labeled arrows. (b) Presence of two intensity peaks in the DLS data suggests the presence of two multimeric states in the EmrE.

**Fig. 3:**
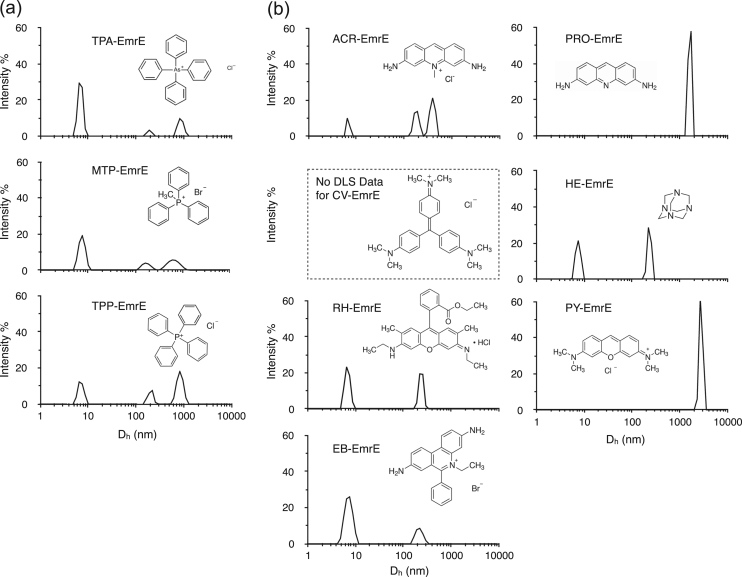

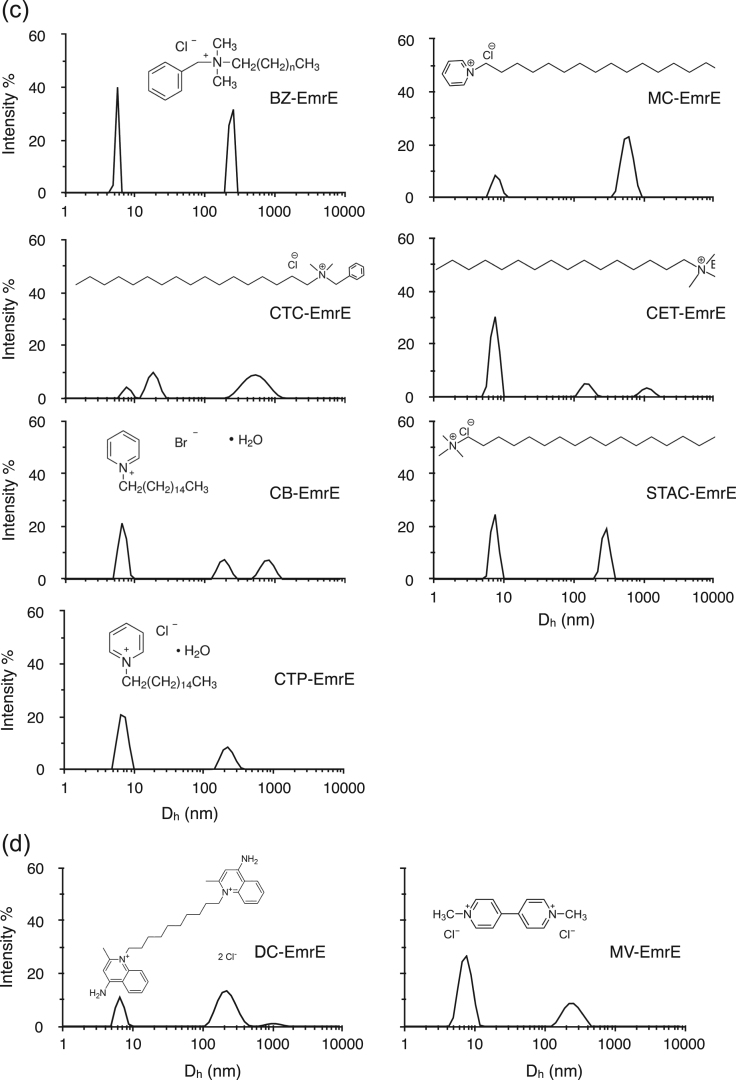
DLS intensity distribution of EmrE in the presence of 19 QCCs. Variations in peak position and intensity suggest the subtle difference in the protein conformation for QCCs tested. Molar ratios for all QCC : EmrE were 1000:1. (a) Sphere-forming QCCs, (b) Poly-aromatic QCCs, (c) Acyl-chained QCCs and (d) Poly-charged QCCs. The results for all QCCs tested are summarized in Table 2.

## 2 Material and methods

### 2.1 Materials

The chemicals used in this study were purchased from either Sigma Aldrich (St. Louis, MO, USA) or EMD Chemicals (Darmstadt, Germany). Electrophoresis equipment and chemicals were obtained from BioRad (Hercules, CA, USA). The DDM detergent, used for protein solubilization and spectroscopy, was purchased from Affymetrix-Anatrace (Santa Clara, CA, USA).

### 2.2 Expression and purification of EmrE

EmrE was expressed from the plasmid pTZEmrEmH6 [25], which contained the *emrE* gene engineered to express with C-terminal Myc epitope followed by a His_6_ tag [25]. Plasmid encoded tagged *emrE* was expressed in the *E. coli* strain C43(DE3) [(F^-^ *ompT hsdSB* (rB^–^ mB^–^) *gal dcm* (DE3)]. Cells were grown in 1 L batches of Luria-Bertani broth (LB, containing 10 g/L tryptone, 5 g/L yeast extract, 5 g/L NaCl). Each 1 L culture batch was inoculated with 10 mL of overnight culture in LB. All cultures contained 0.1 mg/mL ampicillin to maintain the plasmids during cell growth, and were incubated at 37 °C. Growth was monitored by measuring the culture absorbance at 600 nm. When the culture absorbance at 600 nm reached OD_600 nm_ 0.5 – 0.7 (approximately 4 – 5 h), protein expression was induced by adding Isopropyl β-D-1-thiogalactopyranoside (IPTG) to a final concentration of 0.3 mM. The cultures were incubated for an additional 3 h at 37 °C in a shaking incubator. Cells were then harvested by centrifugation at 4000 ×*g* for 10 min at 4 °C and the supernatant was discarded. Harvested cells were stored in re-suspension buffer (20 mM Tris-HCl, pH 8.2, 50 mM NaCl) and frozen at −80 °C. The typical cell yields were approximately 3.3 g/L.

Purified EmrE protein was isolated essentially as previously described [29]. All the steps were carried out at approximately 4 °C. Frozen cell suspensions were thawed and lysed by two passages through a French Press at 10,000 psi. The unbroken cells were separated by centrifugation at 2000 ×*g* for 10 min and the supernatant was collected and subjected to further centrifugation at 120,000 ×*g* for 90 min to collect the membrane pellet. The EmrE-containing membrane pellet, obtained from 6 L of culture, was re-suspended in 25 mL of membrane solubilization buffer (40 mM Tris-HCl, pH 8.2, 100 mM NaCl, 4% (w/v) DDM, 10 mM 2-mercaptoethanol). Re-suspended EmrE-containing membrane fractions were placed in 50 mL falcon tubes and incubated overnight with gentle rocking at 4 °C to solubilize membrane proteins.

The membrane protein re-suspension was diluted 1:1 with distilled H_2_O and centrifuged at 60,000 × *g* to pellet non-solubilized material and the pellet was discarded. NaCl and imidazole were then added to the supernatant to a final concentration of 350 mM and 15 mM, respectively. This sample was loaded onto a 1 mL HisTrap FF immobilized nickel column (GE healthcare, Canada) using ÄKTA purifier (GE healthcare) FPLC system. After loading, the column was washed with 20 column volumes (CV) of wash buffer (20 mM Tris-HCl, pH 8.3, 400 mM NaCl, 65 mM imidazole, 0.1% w/v DDM, 5 mM 2-mercaptoethanol) to remove non-specifically-bound proteins. After washing, EmrE protein was eluted with 10 CV of elution buffer (20 mM Tris-HCl, pH 8.3, 25 mM NaCl, 200 mM imidazole, 0.1% w/v DDM, 5 mM 2-mercaptoethanol).

EmrE was eluted as a single peak and the fractions were pooled together and then injected into a 5 mL HiTrap desalting column (GE healthcare, Canada) to remove the imidazole. The column was already equilibrated with DDM buffer (20 mM Tris-HCl, pH 7.5, 150 mM NaCl, 0.08% w/v DDM) and the sample was exchanged into this buffer. The concentration of the DDM (0.08% w/v) in this buffer corresponded to 1.6 mM DDM, which is well above its critical micelle concentration (CMC) of ~0.2 mM. All of the eluted fractions were analyzed by SDS-Tricine polyacrylamide gel electrophoresis (SDS-T-PAGE; using 12% acrylamide) to confirm the presence and purity of the collected protein. The presence of EmrE-Myc-His_6_ was confirmed by Western blotting the gels onto nitrocellulose and immunoblotting with a conjugated anti-His_6_ or anti-myc horseradish peroxidase antibody (Life technologies, Canada). Desalted EmrE protein samples were pooled together and stored at – 80 °C prior to use for further experiments.

### 2.3 EmrE protein concentration determination

Prior to all experiments the desalted protein samples were thawed and centrifuged at 14,000 ×*g* for 10 min to remove possible aggregates. The concentration of the purified EmrE was then determined using a modified Lowry Assay [30] containing 1% (w/v) SDS to assist in solubilizing the membrane proteins and using bovine serum albumin as protein standard. The stock concentration of the reconstituted desalted EmrE protein in DDM buffer was determined to be 15 µM.

### 2.4 SDS-Tricine PAGE analysis of the EmrE

DDM solubilized EmrE was evaluated using SDS-T-PAGE to identify its multimeric forms according to their predicted molecular weight (MW). During gel casting, trichloroethanol (TCE) was added to a final concentration of 0.5% (v/v) to enable visualization of Trp residues within each protein sample. TCE visualization was performed using UV irradiation at 302 nm as described by Ladner et al. [31]. This in-gel TCE staining technique increased the visibility of EmrE protein by 62% in comparison to conventional Coomassie staining [32]. The EmrE samples, separated by SDS-T-PAGE, were prepared in the incubation buffer (12% w/v SDS, 30% v/v glycerol, 0.05% CBB, 150 mM Tris HCl pH 7.0, 100 mM DTT) from frozen stock samples of the proteins. The samples were mixed by stirring and incubated at room temperature for 30 min prior to loading onto gels. Sample loading volumes for SDS-T-PAGE were typically around 20 µL and the electrophoresis was carried out for approximately 5 h using a voltage range between 40 and 90 V. BioRad low range molecular weight (LMW) standards were used on SDS-T-PAGE. Typically, 1.4 µg of purified EmrE protein was used for each electrophoresis experiment.

### 2.5 Dynamic light scattering

Measurements of dynamic Light Scattering (DLS) from dispersed EmrE with and without QCCs were performed in the Nanoscience Lab (NANS) at the University of Calgary. A separate set of measurements from the control samples (DDM buffer and DDM+QCCs) were also made. Data were collected using a Zetasizer Nano-ZS particle/molecular size analyzer (Malvern instruments) with a He – Ne laser light source that was set to a wavelength of 633 nm at a power of 4.0 mW. Three datasets were collected from each sample and the size distributions of the EmrE in terms of intensity averages were obtained using Malvern instruments software V 7.02. A quartz cell with a 10 mm path length was used to evaluate sample volumes of 300 µL. EmrE samples were prepared in DDM solution buffer at 1000:1 M ratios of QCC to EmrE, and the temperature was maintained at 25 °C during all experiments.

During DLS experiments, using the intensity autocorrelation function, the relaxation rate, Γ, can be extracted and used to determine the translational diffusion coefficient, D, of the particles (EmrE in this study) using the relation D = Γ /Q^2^. Q is the magnitude of the scattering vector given by:

(1) Q=(4πn/λ)sinθ

Where *n* refers to the refractive index of the solution, λ is the wavelength of the scattered light, and 2θ is the scattering angle. The viscosity of the water was taken as 8.9 × 10^−4^ Pa.s and its refractive index as 1.33 at the measurement temperature of 25 °C. The diffusion coefficients of the dispersed particles can be determined from the intensity of the autocorrelation function measured by DLS experiments. Hydrodynamic diameter, D_h_, can then be calculated from the diffusion coefficients, D, by using the Stokes-Einstein relation:

(2) $$D_{h}={(k}_{B}T)/3\pi \eta D$$

Where k_B_T is the thermal energy and η is the viscosity of the dispersion medium. For the dispersions with the presence of multiple species, a regularized fit to the DLS data was applied, since it gives details on the size distribution of the dispersed particles. All the values, presented in this study, were obtained using the software (V 7.02) provided by the Malvern instruments.

### 2.6 Fluorescence spectroscopy

Fluorescence spectroscopy data were collected using a Fluorolog-spectrofluorimeter (Horiba Scientific). Proteins were solubilized in buffered DDM in the presence and absence of QCCs. Sample spectra were collected in a quartz cuvette with 10 mm of the path length at excitation wavelength of 295 nm to specifically excite tryptophans. The emission spectra were measured from 285 to 400 nm using double monochromators for both excitation and emission to reduce scattering artifacts. A 5 nm band pass filter was used for both the excitation and the emission optical paths. The wavelength collection intervals were set at 0.15 nm and for the integration time of 0.1 s. Each collected spectrum was an average of 3 scans per sample. All the fluorescence spectra were collected for three biological replicates (N = 3) and the data averaged. The error bars were calculated as standard deviation over three biological replicates. EmrE was used at a concentration of 1.0 µM. The samples were titrated using stock solutions of QCC in DDM buffer for QCC : EmrE molar ratios of 0.01–1000. All the samples were equilibrated for 10 min prior to the data collection after addition of the respective QCC aliquot. The samples were continuously mixed using a magnetic stir bar at room temperature during the experiments. A separate set of experiments was also performed to ensure that all the fluorescence intensity reductions were due to quenching, wherein the DDM buffer was added instead of QCC solution. All fluorescence spectra were corrected for background Raman spectra. The fluorescence spectroscopy background data sets were collected from blank solutions in the absence of EmrE for all 19 QCCs. The measurements were made for each titration concentration and at the respective excitation wavelengths in DDM buffer. The background data sets were then subtracted from the relevant QCC : EmrE spectra. The inner filter effects were essentially negligible and were ignored during the calculation.

An excitation wavelength of 295 nm (selectively exciting tryptophan residues) was used to obtain ligand-binding curves for QCCs with increasing molar ratios of QCC : EmrE. The intensity loss in the emission spectra was observed as the ligand/ QCC binds to Trp. The plots of this data versus increasing QCC concentration was fitted using Eqs. (3), (4) to obtain ligand-binding curves:

(3) ΔF/Fox100=(ΔFmax/Fox100)[L]/(Kd+[L])

(4) $$\mathrm{Binding}=B_{\max}\times \left[L\right]/\left(K_{d}+\left[L\right]\right)$$

Where the fluorescence intensity change (∆F) to initial fluorescence intensity (Fo) is a measure of ligand binding giving an apparent dissociation constant (Kd), which is the concentration of ligand to reach half maximal binding. B_max_ relates to the maximum specific binding assuming a one binding site model and assuming all chromophores contributing to the fluorescence are accessible to the ligand [L].

## 3 Results

EmrE was examined in the presence of 19 different QCC ligands. The QCCs were divided into four categories: sphere forming (TPA, MTP, TTP), polyaromatic (ACR, PRO, CV, RH, PY, HE, EB), acyl-chained (BZ, MC, CTC, CET, CB, STAC, CTP), and poly-charged (DC, MV).

### 3.1 QCC binding controls EmrE multimeric state and conformation

To study QCC binding to His6-myc tagged EmrE, we used purified protein prepared in a buffer containing 0.08% DDM. EmrE has been assumed to maintain a native-like conformation in DDM-containing buffers [29]. Fig. 2a shows an SDS-T-PAGE gel of purified EmrE wherein two bands are observed, corresponding to the monomer and the dimer with estimated molecular weights of 14.4 kDa and 28.8 kDa, respectively. Analysis of the intensity of each band indicates that ~90% of EmrE occurs as a monomer and ~10% occurs as a dimer under these conditions. The protein is expected to migrate close to a native state in the SDS-T-PAGE [33]. The presence of two oligomer states agrees with the distribution of two sizes observed by DLS experiments using a protein concentration of 0.1 mg/mL where two peaks are seen (Fig. 2b). As DLS data collection is biased to larger particle sizes we do not extract oligomer ratios from such data. It is important to remember that the size obtained from DLS is not only the protein diameter, but also the added size from the detergent shell and any carry over lipid still associated with the protein.

We used DLS to study QCC binding to EmrE at a saturating ligand to protein concentration ratio of 1000:1, and our results are summarized in Table 2, with representative plots shown in Fig. 3. Intensity distribution in DLS showed two consistent peaks at D_h_ between 6 and 8 and 150–250 nm for all the QCCs tested (only proflavine (PRO) and pyronin (PY) show a single peak above D_h_ = 1500 nm, both are poly-aromatic class). There was variation in the 2nd peak position within each group of QCC. For sphere-forming QCCs, this variation in D_h_ is between 174 and 199 nm whereas for the poly-charged group it is between 227 and 252 nm. For poly-aromatic QCC, the position of the 2nd peak is slightly higher (181 and 236 nm) and the range increase between 155 and 286 nm for acyl-chained QCCs. Peaks in the DLS data (Fig. 3, Table 2) at or above D_h_ = 400 nm were also observed for some of the QCCs tested.

**Table 2:** Dynamic light scattering Intensity peaks of EmrE in the presence of all the QCCs used herein are summarized. Shades of grey color, from lighter to darker, represents monomer, dimer and trimer or higher multimers. Black with white text are values we interpret as aggregates.

^a^ Experiments with this ligand did not allow for scattering data to be collected accurately.

**Fig. 4:**
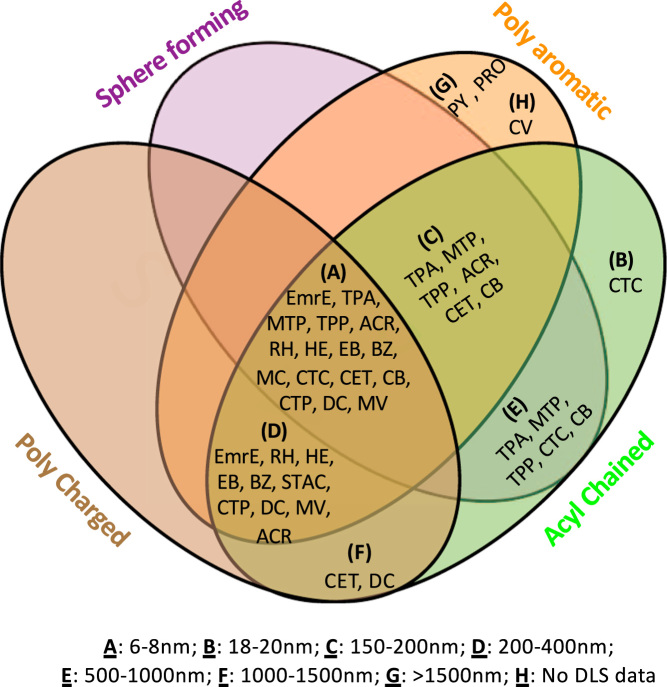
Venn Diagram is generated using DLS data in the Table 2. DLS data were divided into eight size distribution groupings: A, 6–8 nm; B,18–20 nm; C, 150–200 nm; D, 200–400 nm; E, 500–1000 nm; F, 1000–1500 nm; G, > 1500 nm; and H, No DLS data).

The presence of multiple intensity peaks in the DLS data suggests the presence of multiple distinct species and the width of the peak defines the distribution of states in these samples. The Venn diagram in Fig. 4 illustrates the DLS data showing that the conformational shape of EmrE with diameter between 6 and 8 nm is common for EmrE in the presence of almost all the QCCs studied. Position of the second peak from the DLS data of sizes 150–200 nm (C) and 200–400 nm (D) clustered the QCCs into two groups. This implies that the structure of the QCC is influencing different conformational states that affect the hydrodynamic diameter of the EmrE-QCC complex. A few QCCs from acyl-chained and poly-aromatic classes show diameters of the complex above 1000 nm, which are also included in the Venn diagram.

Overall differences are easily observed for the population distributions (width of the peaks) in the plots in Fig. 3(a-d). In a recently published study, results showed large-scale reconfigurations in the structure of EmrE including helical rotation and tilt and repacking of loops [34] upon protonation of Glu-14, a residue considered key in ligand binding. Our global size observations support such changes with ligand binding.

DLS data from the control samples, DDM buffer in the presence of QCCs, but without EmrE, are shown in Fig. S1 in the supporting information. The intensity peak appears at D_h_ ~ 5.2 nm for the DDM buffer. This value is in agreement with those reported for DDM micelles in aqueous solution at pH7 (D_h_ ~ 5.8 nm) [35]. The peak remains between D_h_ ~ 4 and 5 nm with the addition of QCCs. This shows that the addition of QCCs does not significantly affect the DDM micelle size in this buffer. However, for PRO and PY, the peak in DLS data (Fig. S1) appeared at much higher D_h_ value (955 nm for PRO and at 1620 nm for PY). These peaks moved further with higher D_h_ values in the presence of EmrE for both of these QCCs (data for PRO and PY in Table 2).

### 3.2 QCC - Ligand binding reveal different EmrE affinities between different classes of QCC ligands

**Fig. 5:**
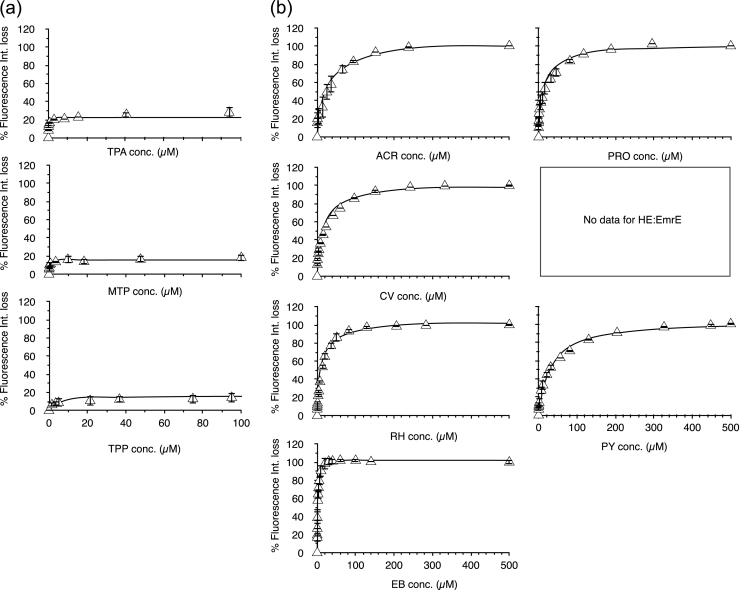

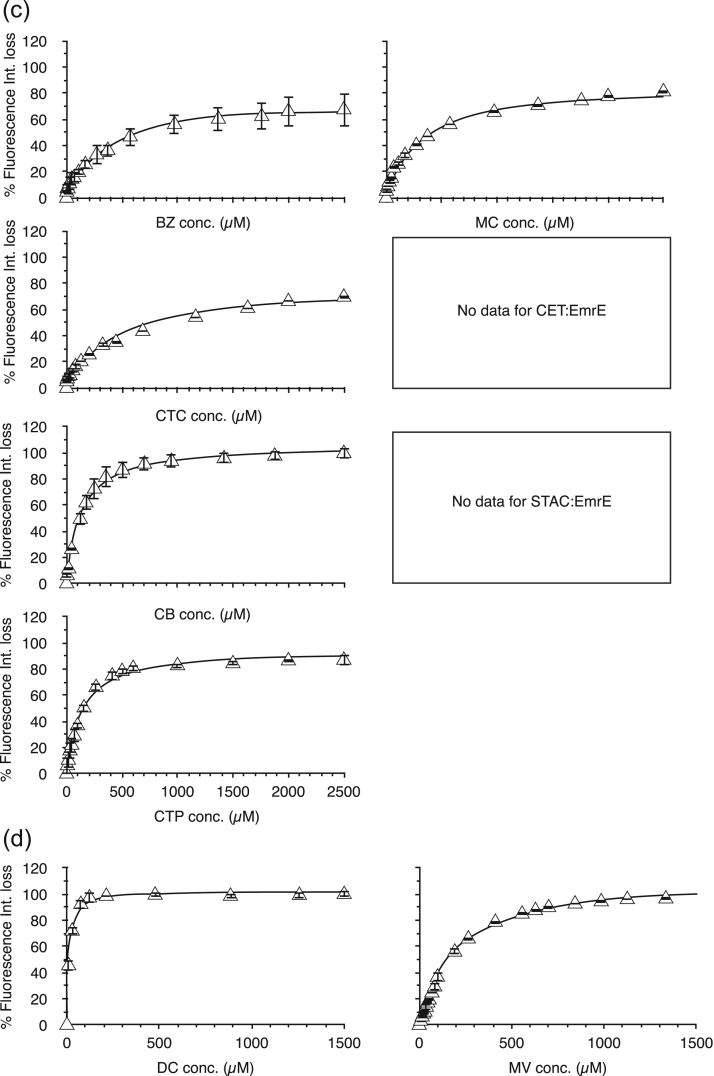
QCC Trp fluorescence quenching spectra of the EmrE. Continuous lines are the fit curves to the data using Eq. (3) in the methods section. K_d_ values and B_max_ values representing the projected end-point of the ligand titration were obtained from the fit curves and provided in Table 3. All QCC titration samples were measured in triplicate (N = 3) and all error bars show standard deviations at each measurement. (a) Sphere-forming QCCs, (b) Poly-aromatic QCCs, (c) Acyl-chained QCCs and (d) Poly-charged QCCs.

Here we used the intrinsic tryptophan fluorescence to monitor ligand binding and to obtain ligand-binding curves. Tryptophan fluorescence is influenced by its environment's polarity/ hydrophobicity/ and dynamics. A change in fluorescence may result from direct ligand interaction with the Trp, and/or conformational change upon ligand binding, or in the case of protein multimerization, from conformational changes arising from protein-protein interactions induced by ligand binding. The present model of EmrE suggests that tryptophan(s) and other aromatic residues are involved in ligand binding [32] and thus we expect that any ligand should lend to a change in tryptophan fluorescence properties; regardless of the ligand's influenced effect on EmrE conformation or multimerization. Regardless of the biomolecular process the changes in Trp fluorescence are dependent on ligand and thus can be exploited to produce binding curves. Fluorescence spectra after background subtraction for EmrE with and without QCC at mid-saturation values for the QCC are shown in Fig. S2. Representative spectra are presented for one selected QCC from each class. Complete fluorescence quenching was observed using poly-aromatic and poly-charged groups of QCCs as shown in Fig. S2. The changes in emission intensity can be plotted against the concentration of ligand (QCC) to determine an apparent dissociation constant (K_d_) for the ligand by following the fluorescence [36], similar to what was done to follow the various drug substrates binding to the multidrug resistance transporter P-glycoprotein [37]. QCC ligand binding curves are provided in Fig. 5(a-d), with curve fits to determine the values of apparent K_d_'s for each QCC interaction with EmrE.

Fluorescence spectroscopy data do not show any changes in the tryptophan fluorescence intensity for HE, CET and STAC. This suggests that there is a lack of binding of these QCC's; although it is possible that such ligands lead to no conformational change or contact with the tryptophan residues, leading to no environmental change for the Trp and thus no fluorescence change. This latter interpretation is likely correct as we observed changes in the DLS in the presence of these ligands.

The data in Table 3 show that there is Trp fluorescence quenching for all other QCCs used in this study. Lower apparent K_d_ values were determined for all sphere forming QCCs, suggesting efficient binding of this class to the protein, whereas the higher K_d_ values for acyl-chained class suggests less efficient binding. The general trend is that the poly-aromatic and poly-charged QCCs have K_d_ values that are an order of magnitude less than that of acyl-chained QCCs.

**Table 3:** A summary of the QCC binding affinities of EmrE determined from Trp fluorescence quenching assays.

**Binding Curves fitting results**			
**QCC**	**Bmax**	**Kd (µM)**	**R^2**
**Sphere forming QCC**			
Tetraphenylarsonium chloride (TPA)	23 ± 2.3	0.1 ± 0.08	0.828
Methyltriphenyl phosphonium bromide (MTP)	16 ± 1.7	0.2 ± 0.09	0.930
Tetraphenylphosphonium chloride (TPP)	17 ± 2.0	5 ± 3.7	0.847
**Poly-aromatic QCC**			
Acriflavine (ACR)	105 ± 16.2	24 ± 13.9	0.914
Proflavine (PRO)	102. ± 5.9	14 ± 5.0	0.941
Crystal Violet (CV)	101 ± 8.1	16 ± 5.7	0.966
Rhodamine 6 G (RH)	104 ± 2.1	12 ± 1.4	0.993
Pyronin Y (PY)	104 ± 3.8	31 ± 5.7	0.984
Hexamethylenetetramine (HE)	No binding^a^		
Ethidium bromide (EB)	103 ± 1.1	1 ± 0.3	0.937
**Acyle-Chained QCC**			
Benzalkonium chloride (BZ)	74 ± 5.6	306 ± 79.5	0.983
Myristalkonium chloride (14 C chain) (MC)	86 ± 5.4	264 ± 59.4	0.987
Cetalkonium chloride (Banjela) (16 Chain) (CTC)	78 ± 7.2	407 ± 133.1	0.979
Cetrimide (CTAB- cetrimonium bromide) (CET)	No binding^a^		
Cetylpyridinium bromide (CB)	107 ± 3.4	128 ± 1.5	0.997
Stearyltrimethylammonium chloride (STAC)	No binding^a^		
Cetylpyridinium chloride (CTP)	95 ± 3.8	137 ± 20.4	0.994
**Poly-charged QCC**			
Dequalinium chloride (DC)	103 ± 2.9	11 ± 2.2	0.996
Methyl Viologen (MV)	111 ± 2.3	179 ± 19.9	0.996

a There was no change in fluorescence intensity with this ligand binding and thus could not determine binding parameters with this approach.

Some of the ligands are chromophores themselves, which could lend to experimental artifacts in the titrations. Thus we tested for inner filter effects, FRET, and also for scattering from detergent vesicles influencing the results. The nature of the format of our fluorometer with double monochromators, selecting both excitation and emission, reduces the scattering effects below the noise of the experimental repeats and thus was not a factor here. Inner filter effects with the dye-based ligands were corrected for by subtraction, and under the conditions used did not influence the data. Additionally, the spectral shape did not change and the emission maximum did not shift more than a few nanometers during the titrations. Thus, the quenching observed is unlikely from solvent accessibility changes. This suggests that all the QCCs affected the Trp environment a similar way and all quench the fluorescence by direct collision or through changing the conformation and/or dynamics of the region around the Tryptophan(s).

Due to the broad nature of the physiochemical properties of different ligands, we still cannot rule out that ligands could influence the spectral changes differently. This is illustrated in those ligands that would be bulkier and sphere like, rather than planar. These ligands gave rise to much lower B_max_ values, between 15 and 22 (Table 3). The B_max_ value of Eq. (4), the value of maximum fluorescence intensity loss (Fig. 5) for the protein can be interpreted as a difference in the available binding. This suggests that the binding of the sphere shaped QCC's is very different from the other ligands. Overall, the fluorescence binding curves demonstrate different levels of affinity and different binding modes of different types of QCCs to EmrE.

## 4 Discussion

The purpose of this study was to compare ligand binding characteristics of a His_6_-myc epitope tagged EmrE over a range of different QCCs ligands. We have previously studied the untagged version of EmrE [11], [13], [25], [32], [38], [39]. However, since the research in other groups use the tagged version, it was important to evaluate this epitope tagged version's QCC ligand binding. We have noted differences between the tagged version and untagged version [25], suggesting the possibility that the tag could influence functionality and oligomer state. Here we evaluated the ligand binding through fluorescence based binding isotherms and size changes by DLS.

EmrE was purified following published procedures involving Ni-affinity chromatography [29] and studied in 0.08% w/v DDM detergent. Purified EmrE was then evaluated for differences in structural size arrangements by DLS and ligand binding by fluorescence spectrophotometry. The differences determined for purified EmrE protein in 0.08% w/v DDM in this study reflect its plastic/ dynamic nature with respect to QCC ligand interaction suggesting that the type of the ligand affects the protein in different ways.

As a control of our system, we performed a SDS-T-PAGE on our preparation of EmrE that showed that both monomers and dimers were present. SDS is not fully denaturing to EmrE and the protein has exhibited ligand binding capability in the presence of SDS [38]. SDS detergent has also demonstrated the ability to solubilize EmrE in a ligand binding competent state [13] and maintains a folding state similar to that observed in DDM-containing buffer [32]. The protein predominantly migrated as monomers, and to a lesser extent as dimers, in agreement with previous studies [18], [25], [32], [39]. The presence of EmrE monomer has been reported in previous experiments that specifically examined EmrE multimerization [25]. Studies of the monomeric EmrE have indicated that the monomer was capable of binding QCC [12], [19], [25], [27]. Although it is important to recognize the multimeric form(s) one is working with, in many publications on SMR proteins only the monomer and dimer are discussed. However, one can often visualize higher order multimers in the reported PAGE figures [13], [32], [40], [41], yet it is rarely noted. Thus the importance of the multimeric form(s) is often ignored when performing biochemistry on detergent solubilized integral membrane protein for the interpretation of the data towards the ‘native’ form in a lipid bilayer.

Here we chose to evaluate the multimeric forms of EmrE using DLS. This approach demonstrated that the protein had two size states in the absence of ligand (Fig. 2 & Table 2). A third size state with higher hydrodynamic diameter (D_h_) was observed in the presence of some QCC ligands. The hydrodynamic diameter, D_h_, for spherical shaped particles is the simplest to estimate from DLS data and it represents the range of the dispersed particles. For other shapes, such as plates, cylindrical particles, prolate or oblate spheres, the D_h_ strongly depends on the long axes [42], [43]. In the case of integral membrane proteins, such as EmrE, the protein-detergent complex includes a shell of detergent molecules. Studies have shown that a untagged EmrE monomer contains around 103 DDM molecules (DDM binding: 4.3 ± 0.6 g/g of EmrE) [11]. It is possible that the tagged version of this protein has a different number of DDM molecules and significant anisotropy in the shape of the EmrE detergent complex. Because of limited or lack of information on the shape of the protein, and the of a variable number of detergent molecules, the molecular mass of the protein cannot be easily estimated. In our case the D_h_ values found reflect a much higher molecular mass than the actual molecular mass of the EmrE protomer units alone reflecting the detergent shell.

The shape of the protein(s) will have significant effect on D_h_ values. This is observed in DLS data (Fig. 3 & Table 2) as the peak positions vary in the presence of different QCC ligands. This demonstrates different conformational state(s) for the EmrE binding respective QCC(s). The DLS data from the blank runs (Fig. S1 – DDM buffer with no protein) showed the intensity peak for the DDM micelles at 5.2 nm. In the case of DDM solubilized EmrE (Fig. 2b), the first intensity peak is shifted at D_h_ ~ 8 nm, likely representing the protein monomer-detergent complex. In the case of the higher multimeric forms of EmrE, with increased anisotropy in its conformational shape, the effect on D_h_ values will be significant. If the conformational state of the protein were a longer rod shaped or a prolate or oblate ellipsoid with increased anisotropy along one axis, there would be a big change in the D_h_ value. Furthermore, such conformation will be surrounded by a different number of detergent molecules that will affect the hydrodynamic diameter. Taking into consideration the anisotropy in the conformational shape and changing numbers of DDM molecules with EmrE, the apparent D_h_ values can be significantly higher than the actual diameter of the protein, as reflected from DLS data in Table 2. As expected, these effects on D_h_ would be prominent for higher multimeric states if the anisotropy increases. This could considerably affect the D_h_ values in higher multimeric states. Irrespective of these limitations, the nature of the QCC can affect the anisotropy in the EmrE multimers and we observe this as differences in peak positions and peak widths. Clustering of tested QCCs in the Venn diagram based on the DLS data (Fig. 4) provides an overview on how the chemical shape of the QCC dictates a conformational/multimeric size for EmrE; and such changes are different for different physic-chemical groupings of QCC substrates.

Understanding the difficulty in quantitatively interpreting DLS data and to avoid the possibility of the presence of larger protein aggregates, the samples were centrifuged prior to all experiments. Hence, the presence of intensity peaks at higher values of D_h_ are not expected from large non-specific aggregates that may form during the sample preparation, but are expected from the higher multimeric forms and different conformational state of the proteins for respective QCC binding. The key observation from our experiments is that the shape and charge of the QCC affects the degree of EmrE conformational and multimeric shape change. This is most frequently seen for the position of the 2nd peak (Table 2) that varies between 156 and 286 nm for different class of QCC ligands. This suggests that the QCCs are causing EmrE to adopt different conformations with unique shapes, possibly anisotropic, surrounded by DDM molecules, which is reflected in the varying peak positions. The longer chain length and amphiphilic nature of some QCCs is also expected to contribute to the anisotropy of the EmrE multimeric state and to a larger variance in the number of associated detergent molecules, and hence in the D_h_ values. DLS data for the sphere-forming QCC class gave three peaks, suggesting multimeric forms higher than dimer. For all QCCs, the monomer peak consistently appeared at the similar D_h_ value with different peak intensities. CV was the only QCC from poly-aromatic class, which did not give DLS data, likely due to the overlap between its absorbance properties and the wavelength of the laser in the DLS.

Although the DLS intensity distribution provides information on the presence and range of sizes in the sample, the scattering caused by bigger particles influences the measurements more, thus causing inherent bias. The scattering intensity distribution must be weighted to account for the bigger particles in the samples containing multiple species. Therefore, it should be noted that the larger intensity peaks at higher D_h_ values are not an indication of a greater amount of large protein/ detergent complexes. Taking this into consideration, the presence of two peaks for the EmrE protein suggested it consists of primarily two different conformational states, and the third peak that appears for only defined classes of QCC ligand, at much higher D_h_ values, may represent even higher multimeric states in EmrE. Large assemblages of EmrE have been observed previously in lipid domain experiments where lipid type and lateral membrane pressure may play a role [44], [45].

Together, the data provide evidence that EmrE protein is highly plastic in shape, oligomeric structure and is capable of transporting a wide range of QCCs. For a limited number of substrates (spherical and planned in shape only) significant structural differences in membrane embedded EmrE are also reported, based on the ligand hydrophobicity, charge and shape [28]. Tetrahedral substrates (spherical shaped in our study) are observed to show correlation between ligand hydrophobicity and binding affinity, which did not hold for planned substrate. This suggests that the charge and shape of the ligand plays an important role for the substrate recognition by EmrE. It was noted that the helix tilt depends on the properties of the substrate and the structural and dynamic changes are likely to be linked [28]. The system presented in our study is different to the membrane embedded protein, being in a detergent. Overall, the structural changes in EmrE to accommodate such a range of substrates with small differences are consistent between these and our studies.

The conformational differences, observed in EmrE in the presence of different QCC ligands are reflected in their relative affinity for particular ligands (Fig. 5 and Table 3). The ligands were selected on the basis of their chemical structure, shape and charge and were divided into four groups/ classes. MV and DC are the only poly-charged QCC, whereas all others tested are monovalent. Poly-charged class is planar in comparison to acyl-chained QCC class, which can act as surfactants. Similar to the poly-charged class, the QCCs in the sphere forming class could shield the central quaternary atom in space filling structures. The binding site of EmrE is located within the transmembrane region which is thought to include the single highly conserved anionic residue, Glu14 [9] that is considered to facilitate both H^+^ and QCC binding [46]. In addition to Glu14, specific Trp residues in EmrE may participate in a cation-pi interaction (as described by [47]), based on Trp mutation studies of EmrE [24].

The structure of the QCC ligand had different effects on the Trp fluorescence quenching that were used to evaluate the apparent K_d_ and B_max_ values listed in Table 3. This data demonstrated that the interaction of the protein with ligand differed for different classes of QCCs tested. For the sphere forming class of QCCs, we observed low B_max_ values (less than 20), suggesting a very different mode of binding compared to other ligands. We also note here that the presence of His_6_-myc affinity tag associated with the construct of EmrE could also occlude binding site(s) and affect the ligand binding; higher B_max_ values were observed for sphere forming and acyl-chained classes of QCC in the untagged version of EmrE in our previous study [25]. The micelle forming nature of acyl-chained QCCs may also affect the quenching process differently and give the slightly lower value of B_max_ for most of the QCCs in this class. For the poly-charged class we observed a B_max_ ~ 100, indicating further that this group binds differently than the other types of QCCs. Within this class, the K_d_ varies in order of magnitude with DC binding strongly to the protein compared to MV. We observe B_max_ of 100 (Table 3) for the poly-aromatic group but with lower values of K_d_ as compared to the acyl-chained group. HE was the only QCC in this group which did not show any change in the fluorescence intensity and thus may not bind to EmrE, whereas, the K_d_ value for EB was an order of magnitude lower in the same group that shows the tighter binding to the EmrE protein. These results suggest that the poly-aromatic ligands may yet have another chemical property separating them.

## 5 Conclusions

This study has revealed further the structural plasticity of small multidrug resistance proteins. We observed structural and binding differences in EmrE for QCCs based on the chemical shape and charge. DLS data groups QCCs based on their influence on the hydrodynamic diameter of EmrE that provides an overview on how the type of QCC dictates a specific conformational state the protein. The results have shown that the type of the ligand affects the shape, multimeric and folding states of the protein. Ligand binding affinity strongly depends upon the physico-chemical properties of the QCC. In general, EmrE is a versatile multi-substrate efflux transporter that can adapt its structure and multimeric state to accommodate a wide range of QCC ligands with a wide range of different structures.
