# Supplementary material for: Influence of quaternary cation compound on the size of the Escherichia coli small multidrug resistance protein, EmrE
Source: Biochem Biophys Rep. 2018 Feb 20;13:129–40. doi: 10.1016/j.bbrep.2018.02.001 (PMC5852267; doi:10.1016/j.bbrep.2018.02.001)
Supplement: Supplementary file 2 — Supplementary material [file mmc2.pdf]

Supporting Information:

Influence of Quaternary Cation Compound on the size of the *Escherichia coli* Small Multidrug Resistance Protein, EmrE

S. Junaid S. Qazi, and Raymond J. Turner

Department of Biological Sciences, University of Calgary, Calgary, Alberta, Canada T2N 1N4

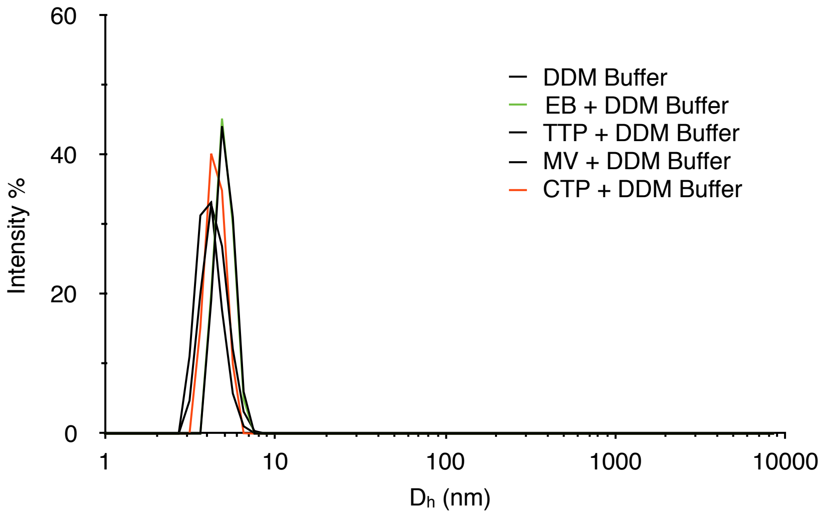

(a)

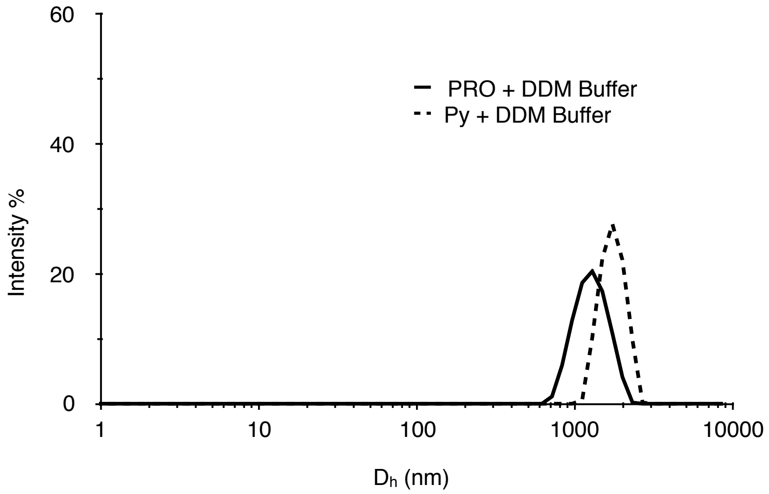

(b)

Figure S1. DLS data, representative from each class is shown in (a). The peaks from the DDM buffer appeared at D_h ~ 5.2nm whereas the position varies between 4 and 5nm in the presence of QCCs. (b) PRO and PY in the DDM buffer, the significant shift in the peaks are observed which further move towards higher D_h values (PRO from 955 to 1620nm and PY from 1281 to 2782nm) in the presence of EmrE (DLS plots in the manuscript).

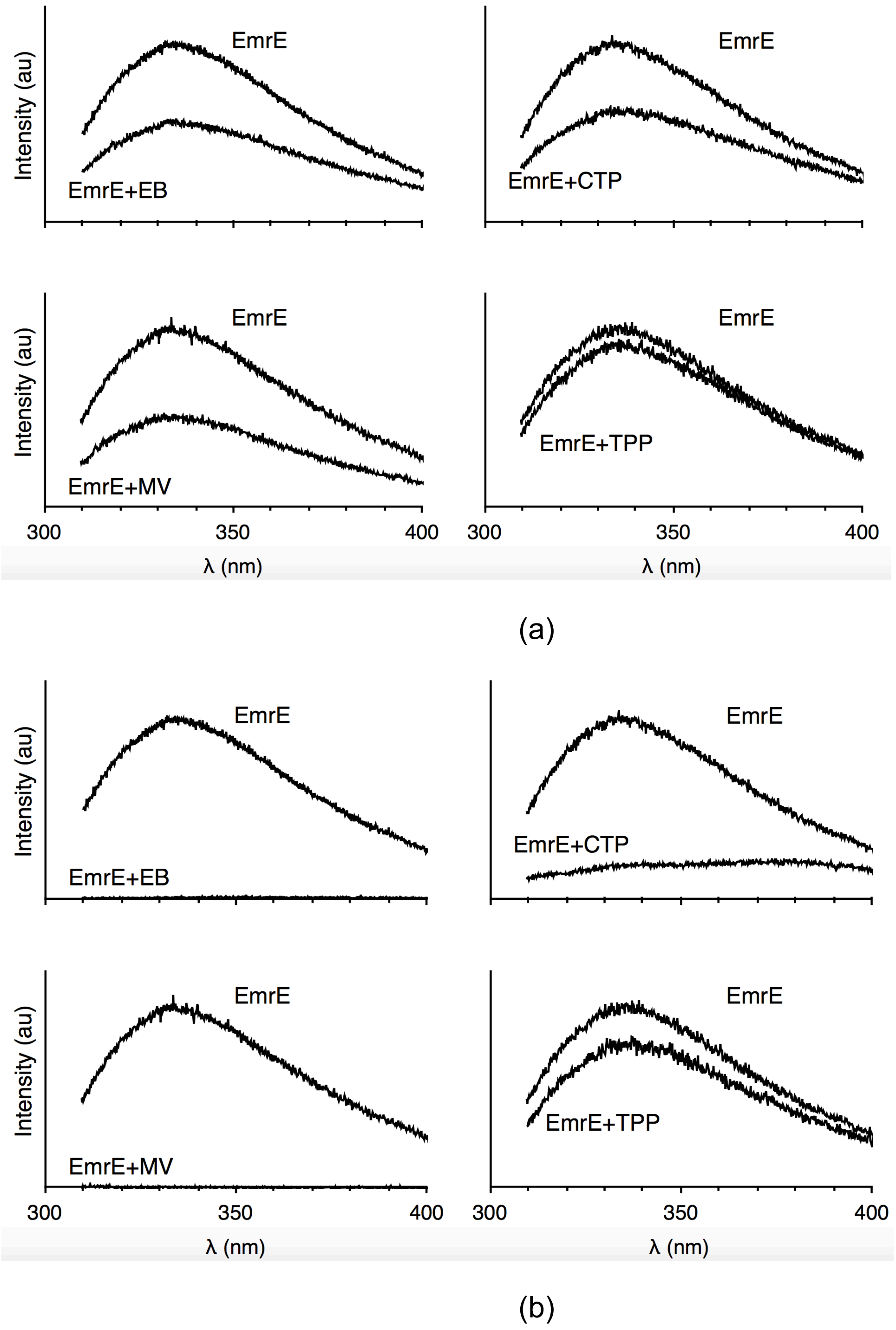

Figure S2. Fluorescence emission spectra from the four QCCs as a representative from each group at the (a) mid-saturation values and at the (b) saturation values of QCCs.
